# Combined effect of thyme and clove phenolic compounds on *Xanthomonas campestris* pv. *campestris* and biocontrol of black rot disease on cabbage seeds

**DOI:** 10.3389/fmicb.2022.1007988

**Published:** 2022-10-28

**Authors:** Eliška Hakalová, Jana Čechová, Dorota A. Tekielska, Ales Eichmeier, Joël F. Pothier

**Affiliations:** ^1^Mendeleum – Institute of Genetics, Mendel University in Brno, Brno, Czechia; ^2^Environmental Genomics and Systems Biology Research Group, Institute of Natural Resource Sciences, Zurich University of Applied Sciences (ZHAW), Wädenswil, Switzerland

**Keywords:** carvacrol, thymol, killing assay, synergistic antibacterial effect, seed treatment

## Abstract

The seed-borne bacterium *Xanthomonas campestris* pv. *campestris* (Xcc) as a causal organism of black rot disease remains the most serious bacterial problem of agricultural production of cruciferous plants worldwide. The eradication of a primary inoculum originating in seeds is available, but no treatment is totally effective. With the threat of developing chemical resistance and increasing pressure for sustainable disease management, biocontrol methods represent one of the main strategies currently applied in agriculture. Natural antimicrobials, including essential oils, are promising tools in disease management with low risks of environmental pollution and impact on human health. Thyme and clove essential oils were demonstrated to be highly effective in *Xanthomonas* studies *in vitro*; therefore, their application in black rot control was evaluated in this study. From five phenolic substances originating from thyme and clove essential oils (carvacrol, eugenol, linalool, *p*-cymene and thymol), the most promising *in vitro* results were observed with carvacrol, for which 0.0195% led to the death of all Xcc cells in 30 min. Moreover, a synergistic antibacterial effect of carvacrol and thymol solutions decreased the minimal inhibition concentration to 0.0049% and 0.0195% for carvacrol and thymol, respectively. Using the quadruple bactericidal values, the complete elimination of Xcc from the surface of infested cabbage seeds was obtained for both carvacrol and thymol solutions and their combined mixture at 2 MIC value. The elimination of bacterial infection from germinated cabbage plants was observed for both plate counting and quantitative real-time PCR methods. We also evaluated the effect of the application of phenolic treatment on the seed germination and germinated plants. Our results suggest a high potential of the application of carvacrol and thymol in vegetable seed production, specifically for cabbage, thus representing a suitable alternative to cupric derivatives.

## Introduction

*Brassica* crops are a large group of plants with a cosmopolitan distribution including many of the world’s most commonly cultivated oilseeds and vegetables (Fahey, 2016). As the main threat of brassicas, black rot disease is reported annually. The disease was considered of relatively minor importance to crucifer production until the 1970s, when approximately 70% of several million transplants were systemically infected from one single seedbed in the United States and losses of 1 million dollars were estimated in 1976. In the 1990s, recurrent black rot epidemics were reported in Italy, India, Japan, and Russia, where the disease caused 23%–90% losses in susceptible *Brassica* cultivars (CABI, 2022). Afterwards, significant losses of production were also reported from Brazil (Peruch et al., 2006) and the Republic of South Africa, where 27% of growers from Gauteng Province presented black rot as the most important disease even when certified seeds were used (Mandiriza-Mukwirimba et al., 2016). In 2019, disease losses of 35.4 thousand dollars were estimated in Georgia, United States (Little, 2021). In the case of hybrid *Brassica* seeds, economic losses of 1.5–8.4 thousand dollars per ha were reported from New Zealand (van Zijll de Jong et al., 2010). The causal agent of the disease is *Xanthomonas campestris* pv. *campestris* (Xcc), a seed-borne bacterium that is among the most important cruciferous pathogens worldwide (Vicente and Holub, 2013; Cruz et al., 2017). Breeding of resistant cultivars is complicated because of the existence of different races and a specific reaction of *Brassica* genotypes to these groups of strains. Currently Xcc is divided into 11 races, among which races 1 and 4 are considered to be prevalent worldwide (Fargier and Manceau, 2007; Vicente and Holub, 2013; Cruz et al., 2017).

Despite considerable efforts to produce Xcc-free seeds, primary infections still occur. Physical treatments are useful to eliminate Xcc from the seed surface but they are not always effective in the case of deep-seated infections that are present in the seed coat, endosperm, or embryo (van der Wolf et al., 2013). Generally, strategies for the prevention and suppression of seed-borne diseases caused by xanthomonads involve the application of physical methods of seed disinfection, such as hot water treatment or dry-heat (Carisse et al., 2000; Scott and Dung, 2020; Pečenka et al., 2021); the use of chemical compounds, such as sodium hypochlorite or copper-based compounds and nanoparticles (Sahin and Miller, 1997; Carisse et al., 2000; Carvalho et al., 2019; Majumdar et al., 2019); biocontrol agents, including antagonistic microorganisms (Lanna-Filho et al., 2013; Liu et al., 2016; Príncipe et al., 2018) or bacteriophages (Obradović et al., 2004; Lang et al., 2007; Balogh et al., 2008); plant extracts and metabolites (Rahman et al., 2008; van der Wolf et al., 2008; Krauthausen et al., 2011; Silva et al., 2013); or the breeding of resistant cultivars. Currently, the main control strategies used in *Xanthomonas* disease management are seed treatment and application of copper and copper-based antimicrobial compounds. These applications are widely used and are still considered the most efficient to control bacterial disease.

Copper itself is required by prokaryotic organisms to maintain a normal cell function. However, above a certain concentration, it is toxic to the cell. Copper antimicrobial activity resides in the production of highly toxic hydrogen peroxide radicals able to weaken membrane integrity when bound (Suwalsky et al., 1998) and to damage DNA (Rodriguez Montelongo et al., 1993). It also substitutes essential ions by redox properties, blocks protein functional groups and inactivates enzymes (Low et al., 2017). The advantages of copper and copper-based compounds are high effectiveness to plant pathogens, low cost, low mammalian toxicity of fixed copper and chemical stability. On the other hand, the development of copper-resistant strains, increased accumulation of copper in soil and its negative effect on a soil microbiome led to the regulatory pressure to limit copper use in agriculture worldwide (Lamichhane et al., 2018). Searching for copper alternatives meeting ecological criteria and efficiency in disease management, combined with a low availability of *in vivo* results, slowed down the implementation of sustainable crop protection strategies by growers.

Compared to the copper substances, organic compounds are well accepted and generally recognized as safe (Lambert et al., 2001). Essential oils (EOs) as a part of the plant response to the microbial invasion have been extensively studied and their antimicrobial properties were documented on a relatively broad range of human, animal, and plant pathogens. However, the detailed knowledge necessary for the application of EOs and their constituents in a practical sphere is still missing. Moreover, most studies are focused on the growth reduction instead of bactericidal activity, which is, in case of plant disease management, of utmost importance (Santos et al., 2022). The active compounds of EOs can be divided into four major groups based on their chemical structure—terpenes (e.g., *p*-cymene, limonene), terpenoids (e.g., carvacrol, geraniol, linalool, menthol, thymol, etc.), phenylpropenes (e.g., cinnamaldehyde, eugenol, vanillin) and a group marked as others representing products originated from fatty acids, lactones, glycosides or sulfur and nitrogen-containing compounds (e.g., allicin, allyl isothiocyanate). From these, terpenes do not represent compounds with high antimicrobial activity, whereas terpenoids and phenylpropenes are described as active against a broad spectrum of microorganisms (Hyldgaard et al., 2012).

The most active monoterpenoids identified so far are carvacrol and thymol. The antimicrobial properties of carvacrol are mainly attributed to the effects on the cytoplasmatic membrane. Its interaction with lipid layer results in the expansion and destabilization of the membrane structure, increased fluidity and permeability and formation of membrane channels for the release of ions, cellular material, ATP and nucleic acid from the cytoplasm (Lambert et al., 2001; La Storia et al., 2011; Nostro and Papalia, 2012). The primary mode of thymol antibacterial action is believed to involve outer and inner membrane disruption, interaction with membrane proteins and intracellular targets, and leakage of potassium ions and ATP similarly to the carvacrol effect (Turina et al., 2006; Hyldgaard et al., 2012). For both compounds, properties to reduce the capability to form biofilm (Nostro et al., 2007; Miladi et al., 2017), decrease bacterial cell motility (Burt et al., 2007; Upadhyay et al., 2012) and inhibition of efflux pumps (Miladi et al., 2016) were also reported.

The antimicrobial activity of phenylpropenes depends mainly on the number and kind of substituents on the aromatic ring. Eugenol, as the best-known member of phenylpropenes, is studied especially for the ability to increase membrane nonspecific permeability, interact with proteins and inhibit the activity of enzymes as ATPase, histidine decarboxylase, amylase or protease (Marchese et al., 2017a).

The main disadvantage of EOs compared to the copper-based compounds resides in their variable chemical composition and poor storability. EOs are complex mixtures of up to 45 different constituents which vary based on the environmental parameters such as geographical location, bioclimate or soil type, season of harvest and also method of extraction (Hyldgaard et al., 2012; Mancianti and Ebani, 2020). Regarding the individual constituents, compounds exhibiting the antimicrobial activity pose a volatile character that affects their application in the disease management. Nevertheless, the inhibitory effect of EOs to *Xanthomonas* genus has been continuously reported during last 15 years, especially in the connection with clove (van der Wolf et al., 2008; Hunag and Lakshman, 2010; Lucas et al., 2012a; Chowdappa et al., 2018; Kumar et al., 2021) or thyme plants (Chudasama and Thaker, 2012; Lucas et al., 2012b; Singh et al., 2017; Chowdappa et al., 2018; Mačionienė et al., 2022). Moreover, the development of resistance to the phenolic compounds was not reported. Even the mechanism of action of both types of antibacterial compounds are similar, contrary to copper, phenolic substances do not represent molecules commonly metabolized during bacterial life cycle and the mechanism for their processing has not been developed yet.

The presented study aimed to analyze an antibacterial effect of the main constituents of thyme and clove essential oils which showed a strong bactericidal effect against bacterium Xcc *in vitro* and evaluate their potential and application in black rot disease management.

## Materials and methods

### Bacterial isolates and growth conditions

Isolates of Xcc were obtained from the National Collection of Plant Pathogenic Bacteria (NCPPB, United Kingdom), Horticultural Research International from the University of Warwick (WHRI, United Kingdom) and Collection of Microorganisms of Mendeleum—Institute of Genetics (MEND, Czech Republic). Based on the host, geographical origin, race and year of isolation, 12 Xcc isolates were used (Table 1). After 24 h growth on Luria Agar (LA, HiMedia, Mumbai, India), the strains were stored in cryovials at −80°C for long-term storage. Before each experiment, the strains were revived on Luria agar and subsequently grown in Luria-Bertani broth (LB, HiMedia, Mumbai, India). Unless stated, the strains were routinely grown on LA plates or in LB broth at 28°C for 24 h in the dark on an orbital shaker (150 rpm, Biosan, Riga, Latvia).

**Table 1:** *Xanthomonas campestris* pv. *campestris* strains used in the study.

Strain^*^	Isolation host	Isolation year	Isolation country	Race
NCPPB 240	*Brassica napus*	1941	United Kingdom	NA
NCPPB 404	*Brassica napobrassica*	1957	New Zealand	NA
NCPPB 528^T^	*Brassica oleracea* var. *gemmifera*	1957	United Kingdom	3
NCPPB 1043	*Brassica oleracea*	1961	Papua N. Guinea	1
NCPPB 1145	*Brassica oleracea* var. *botrytis*	1962	United Kingdom	NA
NCPPB 1685	*Brassica oleracea* var. *capitata*	1965	Australia	NA
NCPPB 1711	*Brassica rapa*	1965	Canada	5
NCPPB 3291	*Brassica oleracea* var. *gongylodes*	1983	Hungary	NA
NCPPB 3927	*Brassica napus* ssp. *oleifera*	1955	Brazil	NA
MEND-B-SU1	*Brassica oleracea* var. *capitata*	2013	Czech Republic	NA
WHRI 1279a	*Brassica oleracea* var. *capitata*	1984	United Kingdom	4
WHRI 3811	*Brassica oleracea*	NA	United States	1

* NCPPB, the National Collection of Plant Pathogenic Bacteria in York, United Kingdom; WHRI, Horticultural Research International from the University of Warwick, Warwick, United Kingdom; MEND-B, Collection of Microorganisms of Mendeleum—Institute of Genetics, Lednice, Czech Republic. A superscript ^T^ at the end of the strain name indicates the type strain of the species. NA, not available.

### Phenolic compounds

Carvacrol, eugenol, linalool, *p*-cymene, and thymol were purchased from p-lab (Prague, Czech Republic). Except for thymol, all phenolic compounds were prepared as 10% stock solutions in LB broth containing 1% dimethyl sulfoxide (DMSO, Penta, Chrudim, Czech Republic) and subsequently serially diluted to the appropriate concentration in the same way. In the case of thymol stock solution, thymol was first dissolved in 96% ethanol (maximum presence of 5% *v*/*v*) and subsequently diluted to the 10% concentration with pure LB broth. Serial dilutions were performed by pure LB broth as well.

### Minimum inhibitory concentration and minimum bactericidal concentration evaluation

For the first evaluation, the strain WHRI 3811 (race 1) was used as the representative of the widely distributed race. Minimum inhibitory concentration (MIC) values were determined by the broth microdilution method in 96-well plates according to da Silva et al. (2019). Briefly, approximately 10^6^ CFU mL^−1^ of *Xanthomonas* strain WHRI 3811 were inoculated into LB broth containing two-fold dilutions of the five individual phenolic compounds (concentration range from 2.5% to 0.005%) in 1:1 ratio. As measurement controls, phenolic compounds diluted by LB broth with 1% DMSO or non-supplemented LB broth were used and the concentration of 10^6^ CFU mL^−1^ of bacterial suspension diluted by pure LB broth served as positive control. Streptomycin solution (0.005%–2.5%) served as an internal control. The optical density after growth was determined at 600 nm (OD_600_) using a microplate reader (SPECTROstar Nano, BMG Labtech, Ortenberg, Germany). All assays were carried out in triplicates. The MIC value was defined as the lowest concentration of antimicrobial agent that inhibited bacterial growth. Values corresponding to the 4 MIC, 2 MIC, MIC, and ½ MIC of three phenolic compounds gaining the highest inhibition effect were tested on other 11 Xcc strains by the broth microdilution method as described above.

The minimal bactericidal concentration (MBC) values were determined by subculturing 5 μL from the broth dilution tests (after 24 h of treatment) on LA without the antimicrobial agent. MBC was defined as the lowest concentration that killed ≥99.9% of bacteria after 24 h of antimicrobial treatment (NCCLS, 1999). All assays were carried out in triplicate.

### Checkerboard titration

The antimicrobial activity of phenolic mixtures was tested *via* a checkerboard titration method according to White et al. (1996). Xcc strain WHRI 3811 at a concentration of 10^6^ CFU mL^−1^ and two-fold dilutions of carvacrol, eugenol and thymol were prepared before mixing. The concentration of each antimicrobial agent ranged from 1/32 MIC to 4 MIC values. The interaction of carvacrol with eugenol and carvacrol with thymol was analyzed by fractional inhibitory concentrations index (FICI), using the following equation:


$$FICI=\frac{MIC\;A\;inmixture}{MIC\;A}+\frac{MIC\;B\;inmixture}{MIC\;B}$$

where *MIC* is minimum inhibitory concentration and *A* represents carvacrol and *B* eugenol or thymol. *FICI* was interpreted as “synergistic” when ≤0.5; as “additive” when >0.5 and ≤1; as “indifferent” (no interaction) when >1 and <4; and as “antagonist” when ≥4. All assays were carried out in triplicate.

### Time-kill assays

The time-kill assay was carried out using the viable cells count method, according to NCCLS (1999), with modifications. Based on the concentrations gaining a bactericidal effect for all 12 tested strains, the Xcc type strain NCPPB 528^T^ was used. Five mL of antibacterial solutions were combined with the bacterial suspension (approximately 10^6^ CFU mL^−1^ based on OD_600_) in 1:1 ratio to reach final concentrations of 0.0195% in case of carvacrol and 0.078% for thymol. Bacterial cultures diluted by pure LB or LB supplemented by DMSO served as growth controls. At nine time points (0, 1, 2, 4, 6, 8, 10, 12, and 24 h) of incubation, 100 μL of cultures were serially diluted and cultivated on LA. The bacterial growth was evaluated after 24 and 48 h.

To establish the minimal exposure time for the seed treatment, the growth of Xcc type strain NCPPB 528^T^ and representative strain WHRI 3811 in permanent contact with the antibacterial compound (½ MIC, MIC, and MBC values) was monitored by the Cell Growth Logger (RTS-1C, Biosan, Riga, Latvia, OD_850_) in 10 min’ period. Bacterial culture of 10^6^ CFU mL^−1^ concentration was combined with carvacrol and thymol solutions in 1:1 ratio to the final volume of 10 mL and cultivated at 28°C and 150 rpm for 48 h. Bacterial cultures without antimicrobial agent served as growth controls.

### Scanning electron microscopy

The morphological changes of Xcc cells treated with carvacrol and thymol compounds were observed using a scanning electron microscopy (SEM; FlexSEM 1000II, Hitachi, Tokyo, Japan). The bacterial suspension of NCPPB 528^T^ at 10^8^ CFU mL^−1^ based on OD_600_ was prepared in LB and separately added to three tubes (5 mL of bacterial inoculum each). The suspension was exposed to carvacrol and thymol solutions (1:1 ratio) to gain final MBC values of 0.0195% for carvacrol and 0.078% for thymol. Untreated suspension diluted by pure LB served as a positive control. After 30 min of incubation at 28°C, each suspension was centrifuged at 5,000 ×*g* for 10 min and harvested cells were washed twice and suspended in sterile water according to Hao et al. (2021). Ten μL of the sample was spread on a carbon sticker, dried in a desiccator and sputtered with gold–palladium (EM ACE 200, Leica Microsystems GmbH, Wetzlar, Germany). The samples were observed with a magnification of 5,000× using an SE detector.

### *In situ* antibacterial activity of phenolic compounds in cabbage seeds and germinated plants

The potential of carvacrol and thymol applications in seed disease management was evaluated by monitoring Xcc presence on the seed surface and in germinated plants. The elimination of a surface infection was observed on a solid medium on Petri dishes. The efficiency of the treatment against the black rot development in young plants and a possible phytotoxicity of selected compounds were observed on germinated plants on filter papers. Regarding the deep infestation of seeds and volatile character of phenolic compounds, the double and quadruple values of the bactericidal concentrations evaluated as effective for all 12 Xcc strains were used in case of pure compounds. For the combined mixture, values evaluated as bactericidal by the Checkerboard titration assay were used.

Seeds of cabbage (*Brassica oleracea* convar. *capitata* L., cv. “Holt”) were surface sterilized and vacuum inoculated with a Xcc suspension (WHRI 3811, 10^6^ CFU mL^−1^ based on OD_600_) according to Roberts et al. (2007). For the negative controls, sterile phosphate-buffered saline (PBS) was used. Seeds were placed into sterile centrifugation tubes (350 seeds per tube) and treated with 2 mL of sterile PBS or carvacrol and thymol in concentrations effective for Xcc elimination *in vitro*. Seed treatment was carried out on an orbital shaker (150 rpm) for 30 min. Untreated seeds inoculated with Xcc served as a positive control, seeds incubated in pure PBS as negative control, seeds treated with individual compounds or their mixtures served as phytotoxicity controls and inoculated seeds treated with PBS as technical control. After the treatment, seeds were air dried 30 min in a laminar flowbox on sterile filter papers. All tested conditions contained 50 seeds per treatment in three repetitions.

The elimination of Xcc from the seed surface was observed by the cultivation of treated and untreated seeds on LA at 28°C and 80% humidity in the incubator (Memmert, Buchenbach, Germany). After 24 and 48 h, the seed contamination index (SCI) was evaluated according to the equation:


$$SCI\;\left[\%\right]=\frac{numberofseedswithobtained\;Xcc\;colonies}{numberoftestedseeds}\times 100$$

where the *number of seeds with obtained Xcc colonies* were determined by the cultivation of colonies from seeds on a selective medium mCS20ABN (Duchefa Biochemie B.V, Haarlem, Netherlands) and by the end-point PCR targeting *hrpF* gene of Xcc according to Berg et al. (2005).

The effect of the treatment to the seed germination and the elimination of Xcc from germinated plants was evaluated according to CISTA (2014) on moisturized filter papers 10 days after the treatment. Germinated plants without testas were homogenized in Bioreba bags (Bioreba, Reinach, Switzerland) with 2 mL of PBS; 100 μL of homogenate was plated in serial dilutions on LA (HiMedia, Mumbai, India) and for the total RNA isolation, 0.5 mL of homogenate and Monarch Total RNA Miniprep kit (NEB, Ipswich, MA, United States) were used. Extracted RNAs were adjusted to 25 ng μL^−1^ and 2 μL were reverse transcribed in the final volume of 20 μL according to Eichmeier et al. (2010). For the real-time PCR assays, Luna Probe qPCR Master Mix (NEB, Ipswich, MA, United States) was used according to manufacturer’s instructions. To evaluate Xcc titer in cabbage tissues, the absolute quantification by the 2^-ΔΔCT^ method (Livak and Schmittgen, 2001) targeting *hrpF* gene of Xcc (Berg et al., 2006) was used. The standard curve was obtained from a serial dilution of a DNA extract obtained from 1 mL of a Xcc suspension with 10^8^ CFU mL^−1^ based on OD_600_, gaining values *R^2^* = 0.99, slope − 3.27 and efficiency of 102%. All reactions were carried out in triplicates in the final volume of 20 μl on the qTOWER^3^ instrument (Analytic Jena, Jena, Germany). For the analyses of results, the qPCRsoft v. 4.0 (Analytic Jena, Jena, Germany) was used.

### Statistical method

The analysis of variant (ANOVA with interactions) and Tukey analysis were used to analyze obtained data and identify the significant differences between evaluated conditions. Values of *p* < 0.05 were considered significant. All statistical analyses were carried out using statistical software package STATISTICA (version 12, StatSoft Inc., Tulsa, OK, United States).

## Results

### *In vitro* antibacterial activity of selected essential oils compounds

Five phenolic compounds of thyme and clove origin were screened for their bactericidal effect to the 12 strains of bacterium Xcc using the broth microdilution assay. For the establishment of initial inhibitory concentration, the representative strain WHRI 3811 was used. The most promising results were obtained for carvacrol, thymol and eugenol with MIC values corresponding to 0.0195, 0.039 and 0.078% solutions, respectively (Table 2). Compared to the antibiotic streptomycin, the minimal bactericidal concentrations of these compounds were at least four times higher.

**Table 2:** Minimum inhibitory concentration (MIC) and minimum bactericidal concentration (MBC) values of studied phenolic compounds for *Xanthomonas campestris* pv. *campestris* strain WHRI 3811.

	Compound [in %]					
	Carvacrol	Eugenol	Linalool	*p*-cymene	Thymol	Streptomycin
MIC	0.0195	0.078	0.156	10	0.039	≤0.005
MBC	0.0195	0.078	0.625	10	0.039	≤0.005

Generally, carvacrol, eugenol and thymol solutions inhibited growth of all Xcc strains used in the study (Supplementary Tables 1, 2). MIC values of individual compounds obtained for the strain WHRI 3811 were effective against all used strains except of the strain WHRI 1279a, where 2 MIC (0.078% solution) was needed to inhibit bacterial growth by thymol (Table 3). The lowest MICs were obtained for carvacrol, when concentrations equal or lower than 0.0098% inhibited growth of eight strains. The 0.0195% solution of carvacrol inhibited growth of the remaining four strains and also proved a bactericidal effect on all treated strains. The most variable results were obtained for thymol, where MICs ranged from values ≤ 0.0195% to 0.078%. In the case of eugenol solution, both MIC and MBC values were from ≤0.039% to 0.078%. Based on these results, the effective concentration for the inhibition of bacterium Xcc was evaluated as 0.0195% for carvacrol and 0.078% for both eugenol and thymol.

**Table 3:** Minimum inhibitory concentration (MIC) and minimum bactericidal concentration (MBC) values of carvacrol, eugenol and thymol solutions for 12 *Xanthomonas campestris* pv. *campestris* strains.

	Carvacrol [in %]		Eugenol [in %]		Thymol [in %]	
	MIC	MBC	MIC	MBC	MIC	MBC
NCPPB 240	≤0.0098	0.0195	≤0.039	≤0.039	0.039	0.078
NCPPB 404	0.0195	≤0.0098	≤0.039	≤0.039	≤0.0195	0.078
NCPPB 528^T^	≤0.0098	0.0195	0.078	0.078	0.039	0.078
NCPPB 1043	0.0195	0.0195	≤0.039	0.078	≤0.0195	0.078
NCPPB 1145	≤0.0098	0.0195	≤0.039	≤0.039	0.039	0.078
NCPPB 1685	≤0.0098	0.0195	0.078	0.078	≤0.0195	0.078
NCPPB 1711	≤0.0098	0.0195	≤0.039	≤0.039	0.039	0.039
NCPPB 3291	0.0195	0.0195	0.078	0.078	≤0.0195	0.078
NCPPB 3927	≤0.0098	0.0195	0.078	0.078	≤0.0195	0.078
MEND-B-SU1	≤0.0098	0.0195	≤0.039	≤0.039	≤0.0195	0.039
WHRI 1279a	≤0.0098	0.0195	≤0.039	0.078	0.078	0.078
WHRI 3811	0.0195	0.0195	0.078	0.078	0.039	0.039

The synergistic antibacterial effect of EOs compounds was evaluated by combining carvacrol with eugenol or thymol and results were estimated by the FIC index method. The lowest concentration of a combined mixture that completely inhibited the growth of Xcc strain WHRI 3811 after 24 h of incubation was 1/16 MIC of carvacrol and ¼ MIC of thymol (Table 4). These concentrations corresponded to the FICI of 0.31. For the combination of carvacrol and eugenol solutions, only additive effect was obtained. Concentrations gaining synergistic effect were subsequently evaluated on all 12 strains used in the study. From three combinations, only the ¼ MIC of carvacrol (0.0049%) and ¼ MIC of thymol (0.0195%) inhibited growth of all tested strains. The bactericidal effect for all strains was observed for their doubled concentration (0.0098% and 0.039% for carvacrol and thymol, respectively).

**Table 4:** *In vitro* effect of carvacrol, eugenol, and thymol combinations on inhibition of *Xanthomonas campestris* pv. *campestris* WHRI 3811 growth.

Carvacrol	Eugenol	Thymol	FICI	Effect
1/2 MIC	1/2 MIC	-	1.00	additive
1/2 MIC	1/4 MIC	-	0.75	additive
1/2 MIC	1/8 MIC	-	0.62	additive
1/2 MIC	1/16 MIC	-	0.56	additive
1/2 MIC	1/32 MIC	-	0.53	additive
1/4 MIC	1/2 MIC	-	0.75	additive
1/8 MIC	1/2 MIC	-	0.62	additive
1/16 MIC	1/2 MIC	-	0.56	additive
1/2 MIC	-	1/2 MIC	1.00	additive
1/2 MIC	-	1/4 MIC	0.75	additive
1/2 MIC	-	1/8 MIC	0.63	additive
1/4 MIC	-	1/2 MIC	0.75	additive
1/4 MIC	-	1/4 MIC	0.50	synergistic
1/8 MIC	-	1/2 MIC	0.63	additive
1/8 MIC	-	1/4 MIC	0.38	synergistic
1/16 MIC	-	1/2 MIC	0.56	additive
1/16 MIC	-	1/4 MIC	0.31	synergistic
1/32 MIC	-	1/2 MIC	0.53	additive

To evaluate the killing kinetics of carvacrol and thymol solutions, the survival of bacterial cells in the presence of antibacterial compounds was assessed by plate count assay at nine time points. Based on a higher susceptibility of the representative strain WHRI 3811 to the thymol solution (MBC value of 0.039%), the Xcc type strain NCPPB 528^T^ was used. The MBC of NCPPB 528^T^ (0.078%) also corresponded to the concentration determined for the effective inhibition of all studied Xcc strains by the thymol solution. However, the strain NCPPB 528^T^ did not provide visible colonies on LA after 24 h incubation and therefore the colony count was done 48 h after the plating on the solid medium (Table 5). The application of carvacrol solution (0.0195%) significantly decreased the number of Xcc living cells compared to the untreated control (supplemented by DMSO), as no colony forming units were detected after 1 h of incubation or at the other seven time points. Moreover, the presence of 0.0195% carvacrol solution provided the bactericidal effect after 30 min of incubation (Table 6). The same efficacy was observed for 0.078% thymol solution (Table 5). Coincidentally, 30 min of the thymol treatment led to the death of all bacterial cells of the NCPPB 528^T^ strain (Table 7).

**Table 5:** Killing kinetics of carvacrol (0.0195%) and thymol (0.078%) to *Xanthomonas campestris* pv. *campestris* NCPPB 528^T^ at nine time points counted 48 h after the treatment [CFU mL^−1^].

Exposure time [in h]	Untreated control (supplemented by DMSO)	Carvacrol (0.019%) + DMSO	Untreated control (non-supplemented)	Thymol (0.078%)
0	5.14 ± 0.57 × 10^5^	4.94 ± 0.16 × 10^4^	1.15 ± 0.06 × 10^6^	0.98 ± 0.15 × 10^6^
1	5.17 ± 0.23 × 10^5^	0	1.56 ± 0.06 × 10^6^	0
2	5.40 ± 0.22 × 10^5^	0	1.59 ± 0.08 × 10^6^	0
4	6.67 ± 0.15 × 10^5^	0	2.18 ± 0.23 × 10^6^	0
6	6.91 ± 0.51 × 10^5^	0	6.37 ± 0.12 × 10^6^	0
8	1.02 ± 0.04 × 10^6^	0	1.14 ± 0.05 × 10^7^	0
10	6.53 ± 0.06 × 10^6^	0	+	0
12	+	0	+	0
24	+	0	+	0

+ presence of *Xanthomonas campestris* pv. *campestris* colonies, uncountable.

**Table 6:** Number of living bacterial cells in 1 h period from the exposition of *Xanthomonas campestris* pv. *campestris* NCPPB 528^T^ to the 0.0195% carvacrol solution [CFU mL^−1^].

Exposure time [in min]	0	30	40	50	60
Untreated control + DMSO	4.16 ± 0.17 × 10^5^	5.04 ± 0.23 × 10^5^	5.22 ± 0.15 × 10^5^	5.33 ± 0.97 × 10^5^	6.25 ± 0.50 × 10^5^
Carvacrol (0.0195%)	3.76 ± 0.16 × 10^5^	0	0	0	0

**Table 7:** Number of living bacterial cells in 1 h period from the exposition of *Xanthomonas campestris* pv. *campestris* NCPPB 528^T^ to the 0.078% thymol solution [CFU mL^−1^].

Exposure time [in min]	0	30	40	50	60
Untreated control	2.69 ± 0.22 × 10^5^	3.97 ± 0.24 × 10^5^	4.01 ± 0.08 × 10^5^	4.21 ± 0.06 × 10^5^	4.15 ± 0.08 × 10^5^
Thymol (0.078%)	2.09 ± 0.07 × 10^5^	0	0	0	0

**Figure 1:**
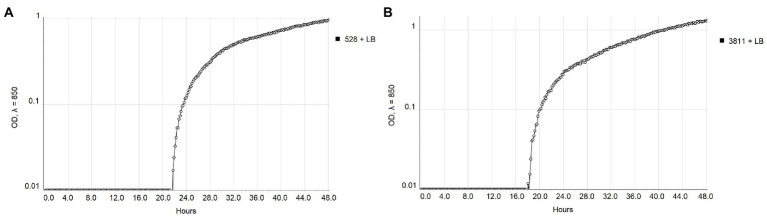
Comparison of the growth of untreated *Xanthomonas campestris* pv. *campestris* strains **(A)** NCPPB 528^T^ and **(B)** WHRI 3811 measured by Cell Growth Logger (OD_850_, 48 h period, log_10_ scale).

**Figure 2:**
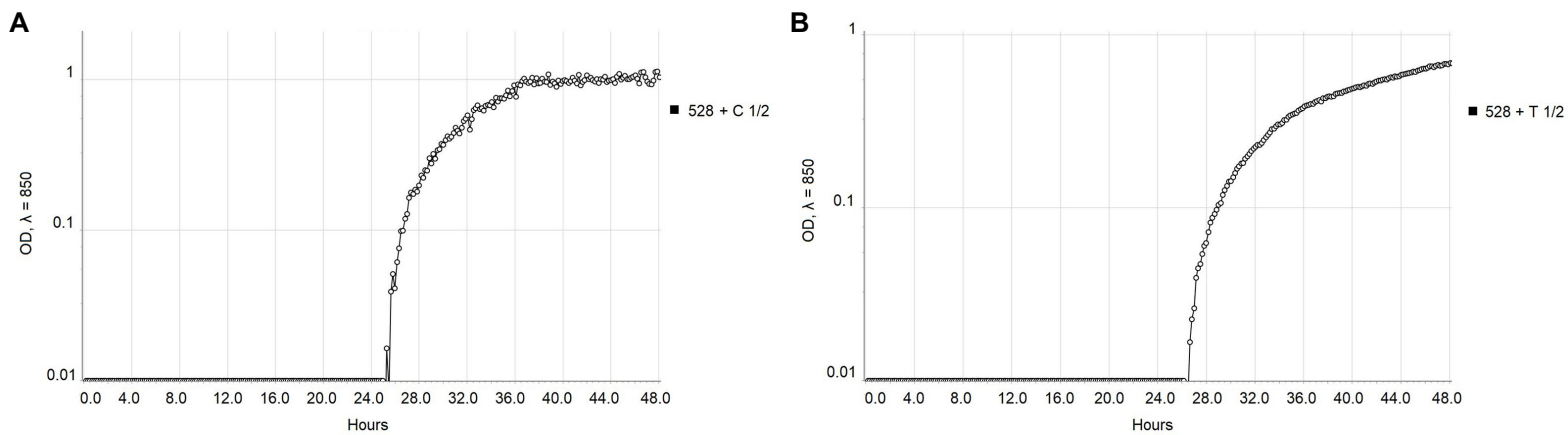
Comparison of the growth of *Xanthomonas campestris* pv. *campestris* NCPPB 528^T^ treated with ½ MIC values of **(A)** carvacrol and **(B)** thymol measured by Cell Growth Logger (OD_850_, 48 h period, log_10_ scale).

**Figure 3:**
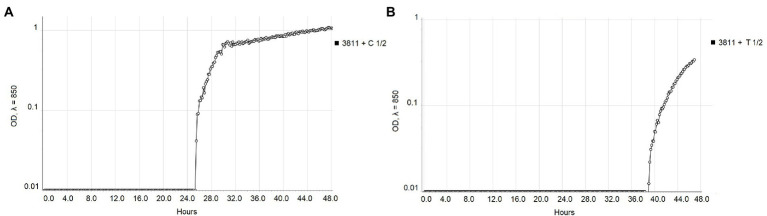
Comparison of the growth of *Xanthomonas campestris* pv. *campestris* strain WHRI 3811 treated with ½ MIC values of **(A)** carvacrol and **(B)** thymol measured by Cell Growth Logger (OD_850_, 48 h period, log_10_ scale).

The real-time monitoring of the change of OD_850_ of bacterial suspension with antibacterial compounds showed differences between strains NCPPB 528^T^ and WHRI 3811. The untreated cultures of these strains differ in the starting time of the exponential growth in almost 4 h (Figure 1). The permanent contact of bacterial suspension with carvacrol and thymol solutions in corresponding MIC and MBC values resulted in the bacteriostatic effect during 48 h of evaluation for both strains (Supplementary Table 3). Nevertheless, a time-dependent bacteriostatic effect was observed for ½ MIC values. Compared to the positive untreated control, the start of the log phase of NCPPB 528^T^ strain was postponed for 3.54 h in case of 0.0049% carvacrol solution and for 4.72 h using 0.0195% thymol solution (Figure 2). The log phase of the Xcc strain WHRI 3811 was slowed down for 7.08 h by 0.0098% carvacrol and 20.57 h by 0.0195% thymol solutions (Figure 3).

**Figure 4:**
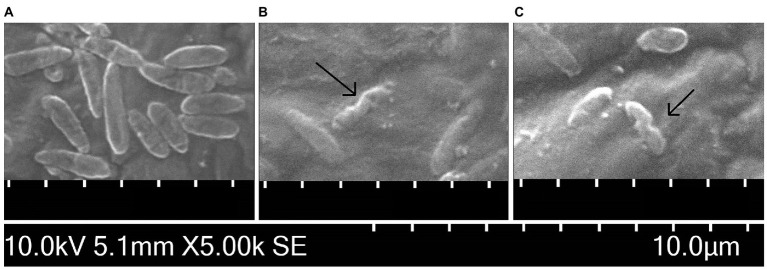
Scanning electron microscopy images of *Xanthomonas campestris* pv. *campestris* NCPPB 528^T^ cells **(A)** untreated, **(B)** treated for 30 min by 0.0195% carvacrol and **(C)** 0.039% thymol solutions. The black arrows indicate alterations of the cell membrane.

The observation by SEM confirmed the ability of both carvacrol and thymol to alter the structure of cytoplasmatic membrane of Xcc NCPPB 528^T^ cells. Figures 4A–C illustrates changes in the Xcc cell morphology compared to the untreated suspension. In the control, rod-shaped, about 2.24 μm long cells with regular and smooth surfaces were observed. Cells treated with 0.0195% carvacrol and 0.039% thymol solutions for 30 min displayed visible deformations of Xcc membrane.

### *In situ* antibacterial activity of carvacrol and thymol solutions on cabbage seeds and germinated plants

Sterile cabbage seeds inoculated by Xcc strain WHRI 3811 were treated with 0.039% carvacrol and 0.156% thymol solutions (double doses of effective bactericidal concentrations) and their mixture consisted of 0.0097% carvacrol and 0.039% thymol in 1:1 (*v*:*v*) ratio. The quadruple doses of concentrations included 0.078% carvacrol and 0.3125% thymol solutions. The evaluation of seeds contamination index (SCI) showed that the growth of Xcc was inhibited in a dose and time dependent manner (Table 8). No colonies were observed for any tested conditions after 24 h of seed cultivation on solid medium except for non-treated positive control and positive control treated with PBS with identical SCI of 96% ± 0.70. Compared to the *in vitro* conditions, the growth of Xcc colonies was observed 48 h after the seed cultivation for the treatments of 0.039% carvacrol and 0.156% thymol represented by SCI values of 97.33% ± 0.70 and 96.67% ± 0.70, respectively. Identity of colonies was subsequently confirmed by both cultivation of presented colonies on mCS20ABN medium and end-point PCR assay. The combined mixture as well as 0.078% carvacrol and 0.3125% thymol solutions exhibited the *in situ* antibacterial activity against Xcc on cabbage seeds surface.

**Table 8:** Seed contamination index [%] for *Xanthomonas campestris* pv. *campestris* (Xcc) infection, germination of treated seeds [%] and quantification of Xcc in germinated plants.

Seed inoculation	Treatment	Seed contamination index [%]		Germination [%]	*hrpF* transcripts/total Xcc (CFU mL^−1^)
		24 h after treatment	48 h after treatment		
Xcc inoculated	None	96.00 ± 0.70	96.00 ± 0.70	96.67 ± 1.76	3.51 ± 1.86 × 10^3^
	PBS	96.00 ± 0.70	96.00 ± 0.70	97.33 ± 1.76	4.06 ± 2.83 × 10^3^
	Carvacrol (0.039%)	0	97.33 ± 0.70	96.67 ± 1.76	7.25 ± 1.76 × 10^4^
	Carvacrol (0.078%)	0	0	96.67 ± 1.76	0
	Thymol (0.156%)	0	96.67 ± 0.70	96.67 ± 1.76	1.08 ± 0.13 × 10^5^
	Thymol (0.3125%)	0	0	94.67 ± 1.76	0
	0.0098% carvacrol + 0.039% thymol	0	0	95.33 ± 1.76	0
PBS inoculated	PBS	0	0	96.67 ± 1.76	0
	Carvacrol (0.039%)	0	0	98.00 ± 1.76	0
	Carvacrol (0.078%)	0	0	94.67 ± 1.76	0
	Thymol (0.156%)	0	0	92.00 ± 1.76	0
	Thymol (0.3125%)	0	0	94.00 ± 1.76	0
	0.0098% carvacrol + 0.039% thymol	0	0	97.33 ± 1.76	0

The application of phenolic solutions did not show significant differences in the seed germination on filter papers. Seeds inoculated by Xcc suspension did not provide a lower germination percentage than seeds inoculated by sterile PBS as well as the non-treated positive control and technical control (Table 8). Neither 0.039% carvacrol and 0.156% thymol nor their doubled values caused a statistically significant difference of germination percentage on the α level 0.05. However, the germination of seeds inoculated by sterile PBS and treated with thymol solutions (0.156% and 0.3125%) gained lower values (92% and 94%) compared to the negative control (97%).

**Figure 5:**
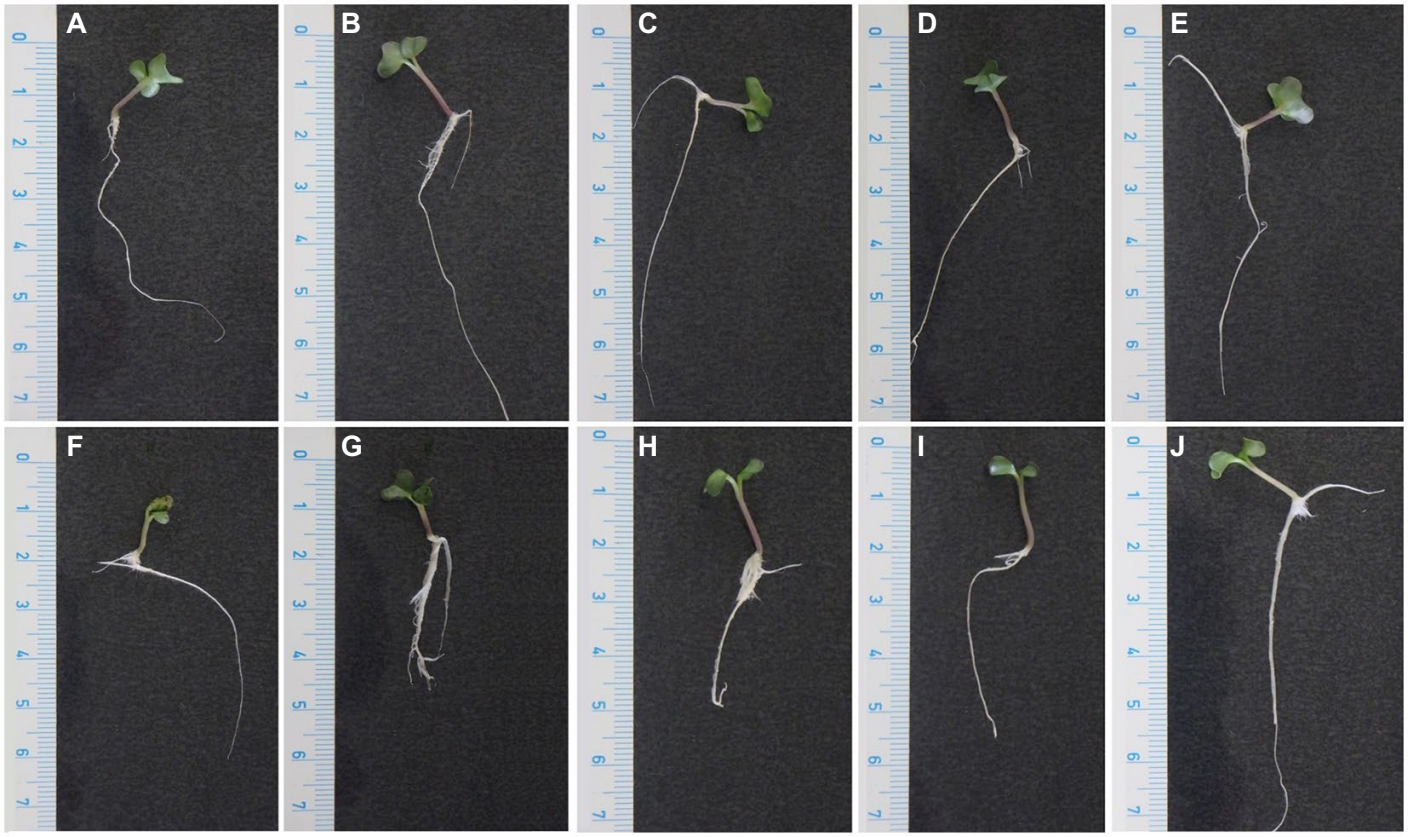
Effect of phenolic treatments on black rot symptoms and cabbage plant vigor. Pictures on top **(A–E)** correspond to 10 days old seedlings obtained from seeds inoculated with sterile PBS and: without further treatment **(A)**, treated with sterile PBS **(B)**, treated with 0.078% carvacrol **(C)**, treated with 0.3125% thymol **(D)**, and treated with a combined solution of 0.0098% carvacrol +0.039% thymol **(E)**. Pictures at the bottom **(F–J)** correspond to 10 days old seedlings obtained from seeds inoculated with a suspension of *Xanthomonas campestris* pv. *campestris* strain WHRI 3811 and: without further treatment **(F)**, treated with sterile PBS **(G)**, treated with 0.078% carvacrol **(H)**, treated with 0.3125% thymol **(I)**, and treated with a combined solution of 0.0098% carvacrol + 0.039% thymol **(J)**.

Plants germinated from seeds treated with phenolic solutions did not show visible deformations of cotyledon leaves or stems (Figure 5). Regarding the manifestation of black rot symptoms, plants from Xcc inoculated seeds treated with solutions of 0.039% carvacrol and 0.156% thymol showed typical small black spots on the cotyledon leaves suggesting an ineffective elimination of bacterial cells from deep pores. However, plants germinated from seeds treated with solutions of 0.078% carvacrol, 0.3125% thymol or with combined solutions showed neither black rot symptoms nor phytotoxic reaction to the phenolic treatment (Figure 5). The presence of visual symptoms was in accordance with results obtained from the cultivation of treated seeds on LA Petri dishes and results of real-time PCR assay (Table 8). The absolute quantification of Xcc was expressed as the number of *hrpF* gene transcripts corresponding to the amount of Xcc (CFU mL^−1^). The qPCR results for samples of cabbage germinated plants from seeds inoculated by Xcc suspension (positive control) and inoculated seeds incubated in PBS (technical control) did not show an influence of the treatment method (30 min soaking) to the reduction of bacterial cells. Interestingly, seeds treated with 0.039% carvacrol and 0.156% thymol showed the increase of detected Xcc cells in almost two orders compared to the both positive and technical control in all three repetitions. Nevertheless, the use of 0.078% carvacrol, 0.3125% thymol and a combined mixture of 0.0097% carvacrol and 0.039% thymol proved the eradication of Xcc from the germinated plants (Table 8).

Based on the germination level and visual condition of germinated plants, an insignificant phytotoxic effect of the used concentrations was evaluated with the application to the cabbage seeds. Moreover, the concentration of 0.078% for carvacrol and 0.3125% for thymol were considered as effective for the elimination of bacterial cells from the seed surface even in case of deep infestation simulated by the vacuum inoculation method. Similar results were obtained for the combined mixture of 0.0098% carvacrol and 0.039% thymol indicating synergistic bactericidal effect of these compounds *in vivo* even in eight-fold dilution.

## Discussion

Diseases caused by plant pathogenic bacteria remain a worldwide threat to food production. Despite the availability of many disease management techniques, the eradication of these diseases still represents one of the most studied areas of agricultural research. Our results showed the ability of five phenolic compounds to inhibit *in vitro* growth of Xcc strain WHRI 3811 (race 1, Table 2). However, the effective dose of *p*-cymene corresponded to the 10% solution. High MIC and MBC values are in accordance with the literature data reported by Marchese et al. (2017b), where *p*-cymene was not evaluated as the main compound conferring the antimicrobial activity of EOs and/or plant extracts. On the other hand, according to Singh et al. (2017) *p*-cymene is able to form potential hydrogen bonds with some residues of catalytic importance by docking in the putative RpfF (regulation of pathogenicity factor F) protein binding pocket of *Xanthomonas oryzae* pv. *oryzae* which indicated that it may play a role in the antibacterial activity of EOs.

The most promising results were obtained for carvacrol, eugenol and thymol solutions. These compounds subsequently proved the inhibition of Xcc growth and also bactericidal activity on 11 Xcc isolates that differed in the host, country and race membership. The highest effectiveness was observed for carvacrol, where MIC and MBC achieved maximum values of 0.0195% representing content of active substance of 0.195 mg mL^−1^ (Table 3). Similar concentration was observed by da Silva et al. (2019), when 0.25 mg mL^−1^ of carvacrol provided both inhibition and bactericidal effect to the Xcc strain Xc-629IBSBF *in vitro*. Close values were also observed by Amini et al. (2018) for eight Xcc local strains from Iran and the strain DSM 1706 (=ATCC 13951), which were inhibited by carvacrol concentration of 122 μg mL^−1^. In case of thymol, both mentioned studies achieved bactericidal effect by lower concentrations, i.e., values of 0.25 mg mL^−1^ (da Silva et al., 2019) and 100 μg mL^−1^ for locals and 50 μg mL^−1^ for DSM 1706 strain (Amini et al., 2018) compared to the prevailing concentration of 0.78 mg mL^−1^ observed in our study. The difference in thymol bactericidal activity may originate in the year of strains’ isolation, geographical origin, or race but the effect of these factors was not observed on the range of isolates used in our study. However, only 12 strains were investigated. Regarding the mechanisms of thymol action, the interaction with cell membrane should bring more similar results, so that the involvement of interaction with other intercellular targets leading to the bacterial cell death is presumed in Iranian strains.

Unfortunately, the study of da Silva et al. (2019) continued only with the original *Lippia gracilis* essential oil (LGRA-106, thymol 60.47%, *w/v* and carvacrol 0.38%, *w/v*) and Amini et al. (2018) with *Zataria multiflora* essential oil (ZMEO, carvacrol 32.51%, *w/v* and thymol 24.59%, *w/v*) and did not evaluate individual effects of carvacrol and thymol in following observations *in vitro* and/or *in vivo*. However, results of growth kinetics showed that LGRA-106 caused the rapid death of Xcc-629IBSB when the viability of cells was reduced after 5 min exposure. Interestingly, for the ¼ and ⅛ MIC the increase in the growth of Xcc was observed (da Silva et al., 2019). Our values obtained by growth logger (OD_850_) for ½ MIC values of tested compounds applied to the both Xcc strains did not show a substantial change in optical density after 48 h evaluation compared to the untreated controls and only a delay in the beginning of exponential growth was documented (Figures 2, 3). In Amini et al. (2018) the effect of emulsion from ZMEO (463.5 μg mL^−1^) was tested also *in vivo* on *X. campestris* contaminated seeds. The 30 min exposure of ZMEO to the pure *X. campestris* suspension led to the complete suppression of bacterial growth and no viable cells were identified. However, 1 h of the seed treatment did not disinfect seeds completely, as bacterial contamination was identified in many germinated seedlings. Similarly to our study, the treatment did not have an effect on the seed germination.

The *in vitro* and also *in vivo* evaluation of antibacterial properties of pure carvacrol on *Xanthomonas perforans* was performed by Qiao et al. (2020) resulting in approximately a thousand times higher susceptibility of this strain to the carvacrol treatment. Interestingly, only 49.3% reduction of disease severity was reported for a double dose of MBC value *in vivo*. The *in vivo* reduced efficiency of the treatment agrees with the results obtained in our experiments where complete eradication of disease was not achieved for double MBC values and quadruple doses had to be used. The effectivity of the Xcc treatment was most probably influenced also by the laboratory cultivation conditions. Contrary to the greenhouse experiments, stable conditions and eradication of an interference with other microorganisms were set. Nevertheless, conditions out of the soil correspond more to the intended purpose of the phenols application when seeds are stored before sowing or expedition by seed growers rather than directly sown, as commonly done in the research. In case of the direct sowing, the effective doses of phenolic treatment have to be confirmed in another experiment.

The natural synergism occurring between individual compounds of EOs is generally evaluated by the Checkerboard titration assay to partially substitute the potential of composed EO by determination of the most effective combination of major compounds or compounds of different origin. In our study, two combinations were investigated: carvacrol + eugenol and carvacrol + thymol. The combination of carvacrol and eugenol showed only an additive effect in the inhibition of the Xcc strain WHRI 3811, as denoted by the FIC index (Table 4). Similar results were reported with the *E. coli* strain CGMCC 1.487 (FICI 0.75) by Pei et al. (2009). Nevertheless, depending on the used *E. coli* strain, both synergistic (FICI 0.17) and antagonistic (FICI 4) effects were also reported (Gallucci et al., 2009; Bassolé et al., 2010) indicating a strain-dependent response. The evaluation of carvacrol and thymol mixtures showed the synergistic effect for the combination of ¼ MIC of thymol with ¼, ⅛ and 1/16 MIC of carvacrol. A presumptive improvement of thymol activity by carvacrol solution may reside in the similar mode of action of these two monoterpenoids. The synergistic effect of thymol and carvacrol was reported in many studies, e.g., by Pei et al. (2009) for *E. coli* (CGMCC 1.487, FICI 0.75) or Zheng et al. (2013) for a broad spectrum of spoilage bacteria (FICI ≤ 0.31) including genera *Acinetobacter*, *Chryseobacterium*, *Dickeya*, *Enterobacter*, *Klebsiella*, *Pectobacterium*, *Pseudomonas*, and *Stenotrophomonas*.

The eradication of Xcc bacterium from the seeds remains one of the most challenging parts of *Brassica* disease management. The difficulty of the achievement of Xcc-free seeds was demonstrated by values of the seed contamination index and qPCR results (Table 8), where both 0.039% carvacrol and 0.156% thymol effective *in vitro* were not able to eliminate the infection *in vivo.* In the case of SCI, the observed delay in a presence of visible colonies on Petri dishes from 24 to 48 h relates to the presence of Xcc cells deep in the seed coat. Due to the vacuum inoculation used for the seed infection, lower concentrated compounds most probably interfered with the bacterial cells on the seed surface but did not cause the death of cells located deeper in seed pores. Higher concentrated compounds evaporated from a seed surface after the treatment during longer time and bacterial cells released from seed pores by increased humidity in the incubation chamber came to the direct contact with the resting phenolic compounds which caused the death of Xcc cells. There is also a possibility that in case of high concentrated compounds, evaporated gas came to the contact with bacterial cells deeper in pores and cause their damage. The antibacterial effect of vaporous carvacrol and thymol was described for example for the Gram-negative bacterium *Aggregatibacter actinomycetemcomitans* by Wang et al. (2016).

In the practical sphere, the most used control method still represents the hot water treatment (HWT). Pečenka et al. (2021) reported that the use of HWT on artificially inoculated cabbage seeds led to the significant reduction of Xcc cells, but the amount of 2.64 × 10^3^ and 1.81 × 10^3^ CFU mL^−1^ were detected from seedling homogenates when using 50°C and 53°C, respectively. Compared to the HWT, the results observed for 0.078% carvacrol, 0.3125% thymol and the combined mixture suggest that phenolic compounds may improve the production of Xcc-free seeds and seedlings, minimally when used for a disinfection of seeds where a presence of Xcc is suspected. On the other hand, the application of insufficient concentration led to the increase of Xcc population in the germinated plants. Although sterilized seeds were used, more resistant microorganisms were predicted to rest on the seeds. These microorganisms might be subsequently eradicated by phenolic treatment and the elimination of competing microorganisms led to the support of the growth of Xcc cells located deep in the seed coat after germination. These results showed the importance of the *in vivo* observation when the use of organic compounds in a disease management is considered.

## Conclusion

Our results suggest a high potential of the application of carvacrol and thymol compounds in vegetable seed production, specifically for *Brassica* crops. The concentration of 0.078% carvacrol, 0.3125% thymol and the combined mixture of 0.0098% carvacrol and 0.039% thymol were considered as effective to the elimination of Xcc from the artificially inoculated seed. Presented concentrations did not negatively affect seed germination in the case of a 30 min of seed treatment and did not cause visual symptoms of phytotoxic reaction of germinated plants highlighting their application potential in the management of black rot disease.

## Data availability statement

The original contributions presented in the study are included in the article/Supplementary material, further inquiries can be directed to the corresponding author.

## Author contributions

EH, JČ, and AE contributed to conception and design of the study. EH and DT performed laboratory analysis. EH wrote the first draft of the manuscript. EH and JP wrote sections of the manuscript. All authors contributed to the article and approved the submitted version.

## Funding

The authors acknowledge the financial support from the Mendel University in Brno project nos. IGA-ZF/2021-ST2002 and CZ.02.1.01/0.0/0.0/16_025/0007314. JP was supported by the Department of Life Sciences and Facility Management of the Zurich University of Applied Sciences (ZHAW) in Wädenswil.

## Conflict of interest

The authors declare that the research was conducted in the absence of any commercial or financial relationships that could be construed as a potential conflict of interest.

## Publisher’s note

All claims expressed in this article are solely those of the authors and do not necessarily represent those of their affiliated organizations, or those of the publisher, the editors and the reviewers. Any product that may be evaluated in this article, or claim that may be made by its manufacturer, is not guaranteed or endorsed by the publisher.
